# Disruption of the association between drug transporter and actin cytoskeleton abolishes drug resistance in hypertrophic scar

**DOI:** 10.18632/oncotarget.13734

**Published:** 2016-12-01

**Authors:** Linlin Su, Lanqing Fu, Yan Li, Fangfang Yang, Min Zhang, Dahai Hu

**Affiliations:** ^1^ Department of Burns and Cutaneous Surgery, Xijing Hospital, The Fourth Military Medical University, Xi’an, Shaanxi 710032, China; ^2^ Department of Orthopedics, Jingzhou Central Hospital, Tongji Medical College of Huazhong University of Science and Technology, Jingzhou, Hubei 434020, China

**Keywords:** hypertrophic scar, fibroblasts, P-glycoprotein

## Abstract

Hypertrophic scar is characterized by the overgrowth of fibroblasts and often considered as a kind of benign skin tumor, thus chemotherapeutic drugs have been used to treat scars. In view of the similarity, this study aims to investigate whether drug resistance in cancer that contributes to the failure of chemotherapy also exists in hypertrophic scar, and what is the possible mechanism. Fibroblasts derived from hypertrophic scar and normal skin tissues were first compared for their resistance to verapamil and etoposide phosphate. Scar fibroblasts showed stronger resistance to both verapamil and etoposide than normal fibroblasts, also scar fibroblasts expressed more P-glycoprotein and MRP1 than normal fibroblasts. When scar fibroblasts were pre-treated with PSC833 or probenecid, a P-glycoprotein or MRP1 inhibitor respectively, the resistance to verapamil or etoposide was strongly attenuated. Moreover, co-immunoprecipitation revealed more association of P-glycoprotein/MRP1 with actin filaments in scar fibroblasts than normal fibroblasts. The resistance in scar fibroblasts to verapamil and etoposide was almost abolished when pre-treated with latrunculin-A or a specific anti-actin antibody. Taken together, this study suggests that the enhanced expression of drug resistance-related transporters and their increased association with actin cytoskeleton contribute to the resistance to chemotherapeutic drugs in hypertrophic scar. Thus, down-regulating the expession of drug transporters or disrupting drug transporter-actin filament interaction might be novel and effective ways for hypertrophic scar treatment.

## INTRODUCTION

Hypertrophic scar (HS) is often considered as a kind of benign skin tumor because of its excessive fibroblast proliferation. A major challenge in cancer treatment is the resistance of cancer cells to chemotherapeutic agents, which is mediated by several drug resistance-related drug transporters [1–2], particularly P-glycoprotein and multidrug resistance-associated protein 1 (MRP1). P-glycoprotein and MRP1 are overexpressed in various tumors [3–4], they exclude therapeutic drugs out of cells and thus decrease the intracellular drug accumulation. Given that HS shares certain similarity with tumor, this study aims to determine whether drug resistance also exists in HS and whether this is mediated by P-glycoprotein and/or MRP1.

Recent studies have revealed that the interaction between drug transporter proteins and cytoskeleton system plays essential roles in various cellular processes, such as cross-membrane substance trafficking, intracellular protein transportation, as well as cell signaling [5–7]. Of interest, emerging evidences have suggested an important role of the actin-based cytoskeleton in P-glycoprotein-mediated drug resistance in cancer cells [8]. In fact, the organization of actin filaments was involved in the expression and function of P-glycoprotein in osteosarcoma cells that exhibit drug resistance [9]. Cytoskeleton alteration was also found in a human breast cancer cell line that shows drug resistance [10]. Therefore, it is worthy to elucidate whether the interaction between drug transporters, such as P-glycoprotein or MRP1, and actin cytoskeleton would be one of the mechanisms that confer HS resistance.

In this study we have investigated the association between P-glycoprotein/MRP1 and actin, as well as the role of this interaction in P-glycoprotein/MRP1-mediated drug resistance in HS. Our results revealed HS-derived fibroblasts were more resistant to both verapamil and etoposide phosphate than normal fibroblasts. HS over-expressed both P-glycoprotein and MRP1, and the use of P-glycoprotein or MRP1 inhibitor could abolish the drug resistance in HS. Stronger association between P-glycoprotein/MRP1 and actin was found in HS, when latrunculin-A, an actin depolymerization agent, was used to disrupt this association, drug resistance in HS was greatly attenuated. Thus the enhanced P-glycoprotein/MRP1 expression and their association with actin cytoskeleton might be a potential mechanism conferring HS resistance to chemotherapy, while disrupting this association by well-designed chemicals would be a novel approach for HS treatment.

## RESULTS

### Hypertrophic scar-derived fibroblasts were more resistant to both verapamil and etoposide phosphate

**Figure 1:**
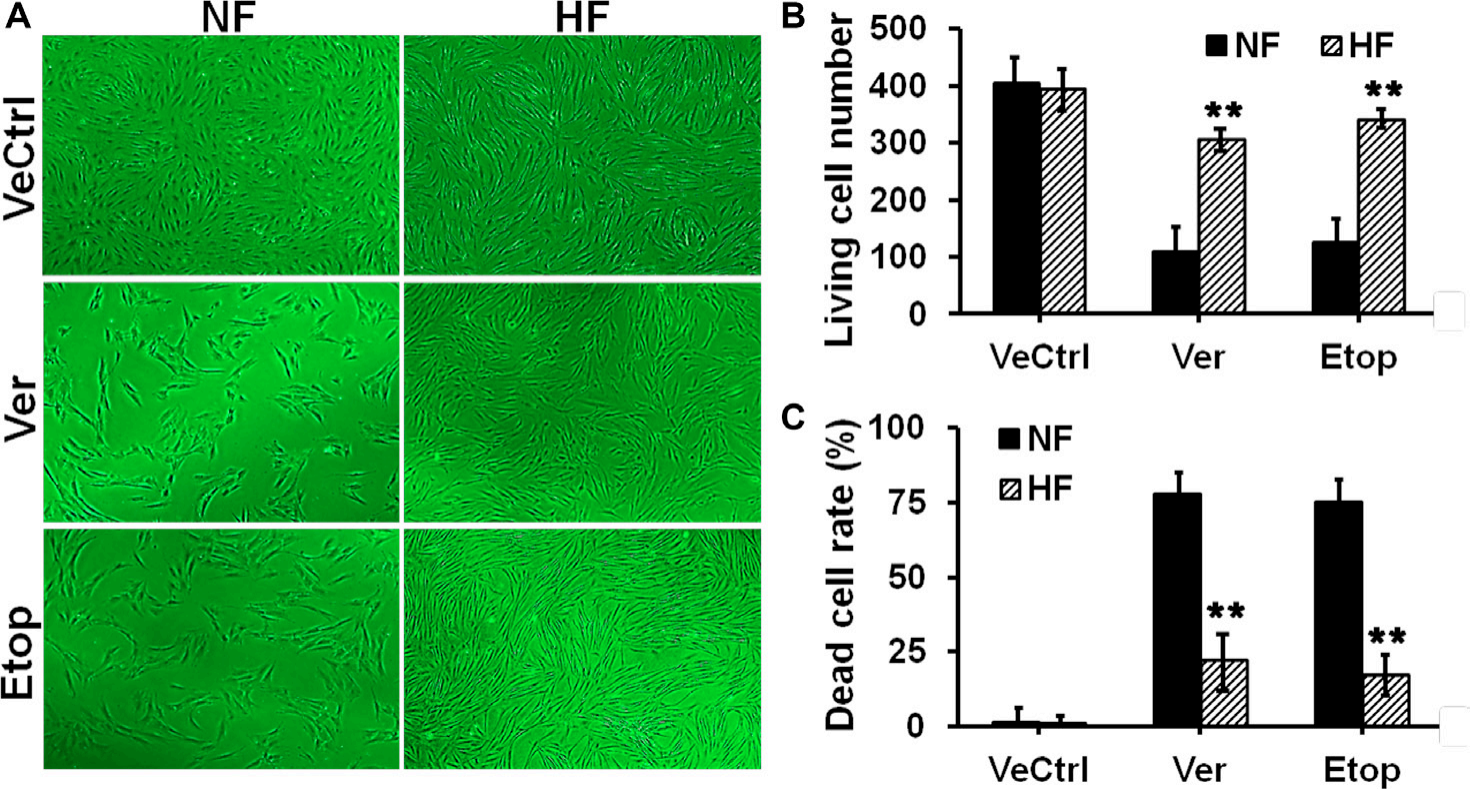
Evaluation of drug resistance in fibroblasts from normal skin and hypertrophic scar (**A**) Normal skin fibroblast (NF) and hypertrophic scar fibroblast (HF) were treated with ethanol (vehicle control, VeCtrl), verapamil (Ver, 200 μM) or etoposide phosphate (Etop, 50 μM) for 12 h, then the cell density in each group was visualized under light microscope. (**B**) The living cell number under each treatment in (A) was counted and compared between NF and HF. (**C**) The dead cell rate in each treatment group was analyzed by flow cytometry and compared. Error bars represent means ± SD (*n* = 4), **p < 0.01.

We first investigated the difference on drug resistance between nomal skin and HS. Fibroblasts from normal skin (NF) and hypertrophic scar (HF) were treated with ethanol (vehicle control, VeCtrl), verapamil (Ver, 200 μM), or etoposide phosphate (Etop, 100 μM) for 12 h, cell survival and death were then analyzed by light microscope (Figure 1A) or flow cytometry (Figure 1C). More living cells were observed in HF than in NF after drug treatment (Figure 1A). Prior to Ver or Etop treatment, there were ~400 living cells in each group, after drug treatment ~300–340 living cells left in HF while only ~100 in NF (Figure 1B). Flow cytometry analysis showed Ver or Etop caused an ~75% cell death rate in NF while only ~23% in HF (Figure 1C), consistent with the observation and counting in Figure 1A–1B.

### The protein levels of P-glycoprotein and MRP1 were up-regulated in hypertrophic scar fibroblasts

**Figure 2:**
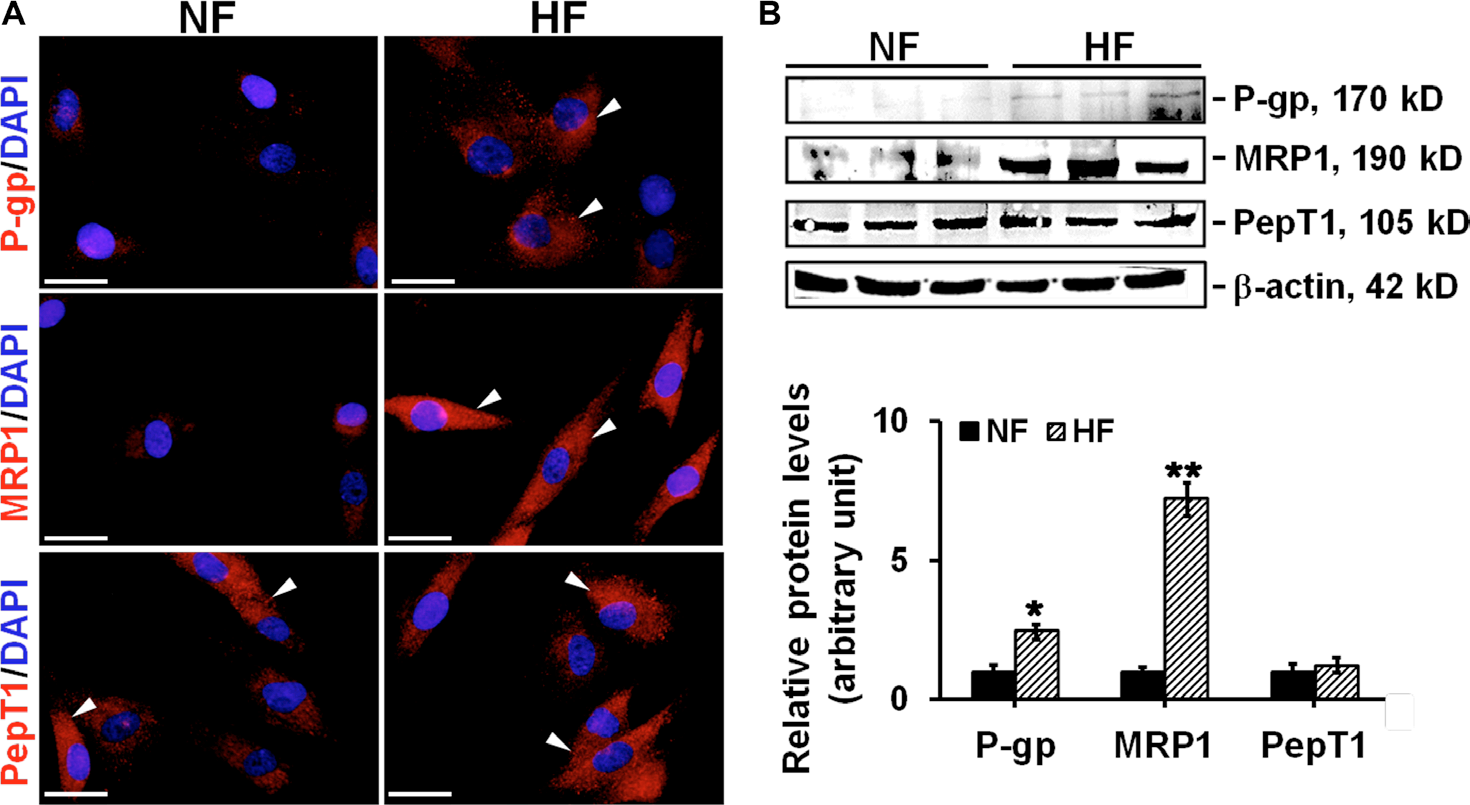
The comparison on the localization and expression of drug transporting proteins in NF and HF (**A**) Study by immunocytofluorescence to assess the cellular localization of P-glycoprotein (*red*), MRP1 (*red*), and PepT1 (*red*) in NF and HF. Nuclei were visualized with DAPI (*blue*). White arrowheads point to the positive immunostainings. Scale bar: 25 μm. (**B**) Immunoblotting of P-glycoprotein, MRP1 and PepT1 in the lysates of NF and HF. β-actin served as an equal protein loading control. Histogram compared the difference on the relative protein expression between NF and HF. Each data point was normalized against corresponding β-actin with the average value after normalization in NF arbitrarily set as 1. Error bars represent means ± SD (*n* = 6), *p < 0.05, **p < 0.01.

Then we studied the expression and localization of two drug resistance-related transporter proteins, namely P-glycoprotein and MRP1, in normal skin and HS. P-glycoprotein and MRP1 showed very few stainings in NF, while exhibited strong cytoplasm distribution in HF (Figure 2A). The protein level of P-glycoprotein or MRP1 in HF was ~2.5- or ~7-fold higher than that in NF, respectively (Figure 2B). PepT1, an oligopeptide transporter that does not confer cell drug resistance, showed no difference on the staining pattern or expession level between NF and HF and served as an internal control in this study (Figure 2A–2B).

### The protein levels of P-glycoprotein and MRP1 were up-regulated in hypertrophic scar dermis *in vivo*

**Figure 3:**
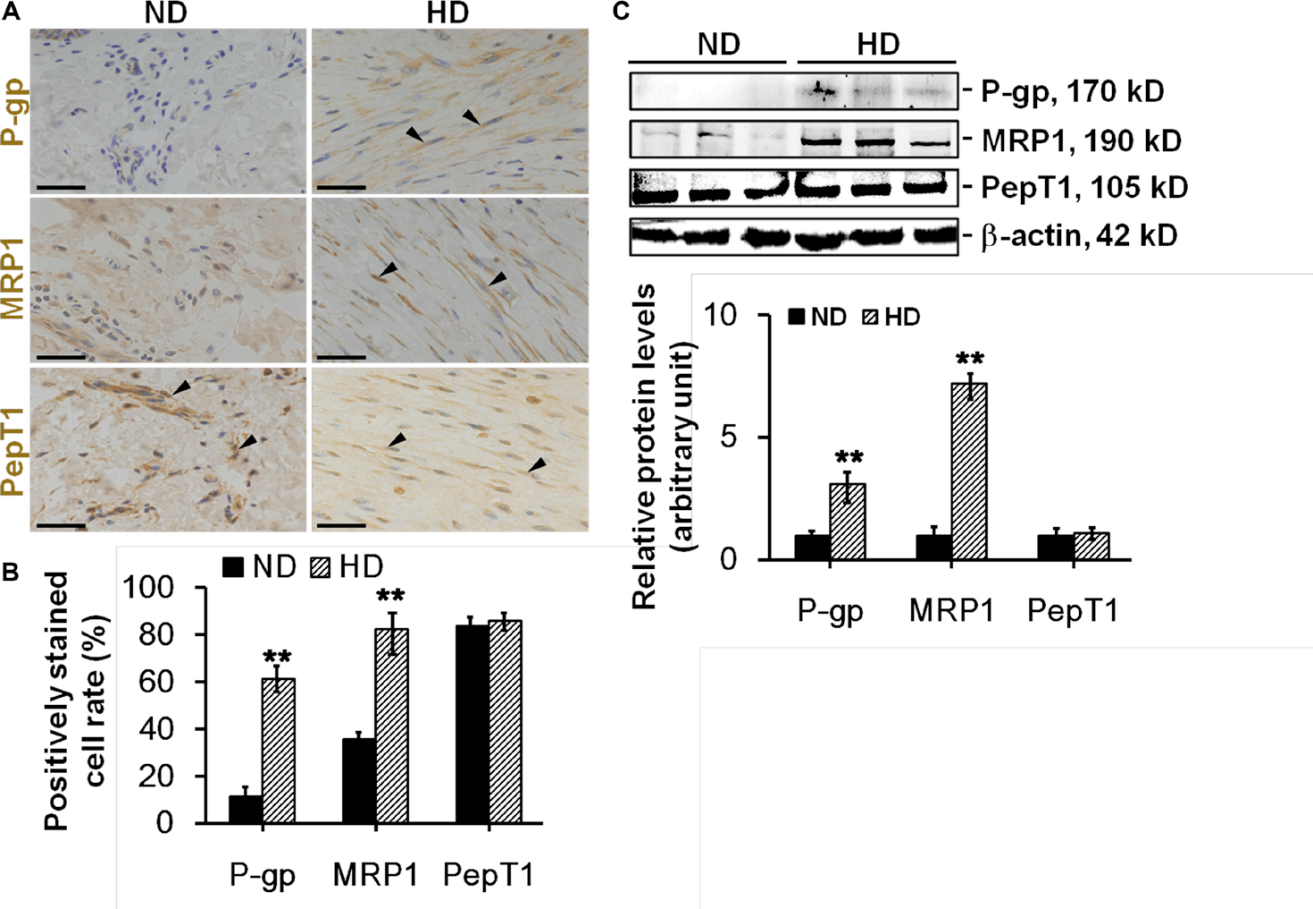
The comparison on the distribution and expression of drug transporting proteins between normal dermis and scar dermis in vivo (**A**) Study by immunohistochemistry to assess the distribution of P-glycoprotein (*brownish*), MRP1 (*brownish*), and PepT1 (*brownish*) in normal dermis (ND) and hypertrophic scar dermis (HD). Nuclei were conterstained with hematoxylin (*blue*). Black arrowheads point to the positive immunostainings. Scale bar: 50 μm. (**B**) Histogram comparing the P-glycoprotein-, MRP1-, or PepT1-positively stained cell rate between ND and HD. (**C**) Immunoblotting of P-glycoprotein, MRP1 and PepT1 in the lysates from ND and HD. β-actin served as an equal protein loading control. Histogram compared the difference on the relative protein expression between ND and HD. Each data point was normalized against corresponding β-actin with the average value after normalization in NF arbitrarily set as 1. Error bars represent means ± SD (*n* = 6), **p < 0.01.

*In vivo* experiments were performed to validate above *in vitro* data. Dermis from normal skin (ND) and hypertrophic scar (HD) were sectioned and stained with anti-P-glycoprotein, anti-MRP1 or anti-PepT1 antibody for immunohistochemistry (Figure 3A–3B). Results showed only ~10% P-glycoprotein or ~35% MRP1-positively stained cells in ND, while ~60% P-glycoprotein or ~80% MRP1-positively stained cells in HD (Figure 3A–3B). Immunoblotting analysis further confirmed above observation, the protein levels of P-glycoprotein and MRP1 were up-regulated by ~3- or ~7-fold respectively in HD compared to ND (Figure 3C). *In vivo* PepT1 distribution or protein expression showed no difference between the two groups (Figure 3A–3C).

### Pre-treating cells with P-glycoprotein or MRP1 inhibitor abolished HS drug resistance *in vitro*

**Figure 4:**
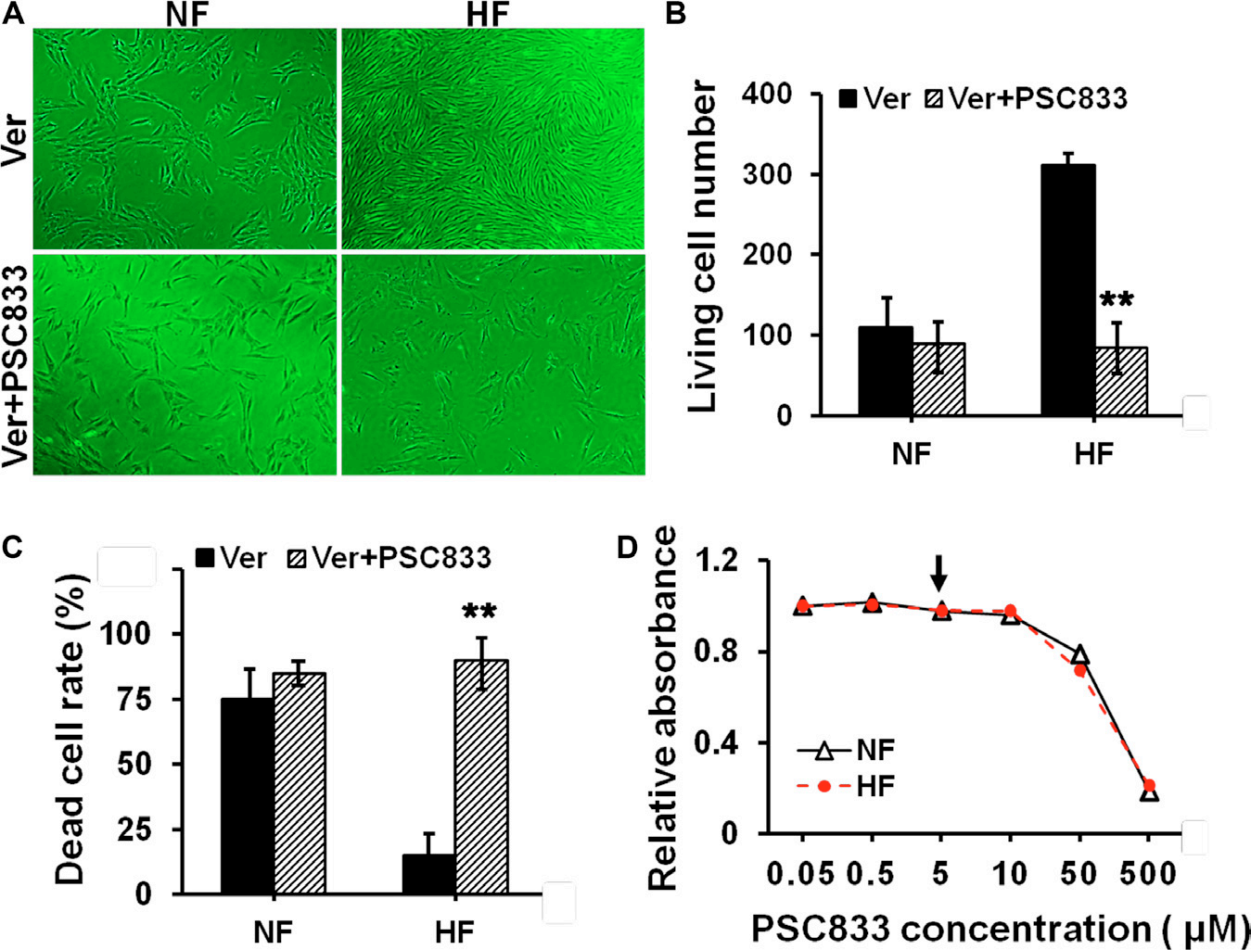
Evaluation of the resistance to verapamil after pre-treating cells with P-glycoprotein inhibitor in NF and HF (**A**) Cell density in NF and HF after Ver alone or with PSC833 pre-treatment was visualized under light microscope. (**B**) The living cell number in each treatment in (A) was counted and compared. (**C**) The dead cell rate in each treatment group was analyzed by flow cytometry and compared. Error bars represent means ± SD (*n* = 4), **p < 0.01. (**D**) The cytotoxicity of PSC833 in NF and HF was determined by MTT assay with the absorbance measured at 540 nm. PSC833 at 5 μM was the concentration used in this study as indicated by black arrow.

**Figure 5:**
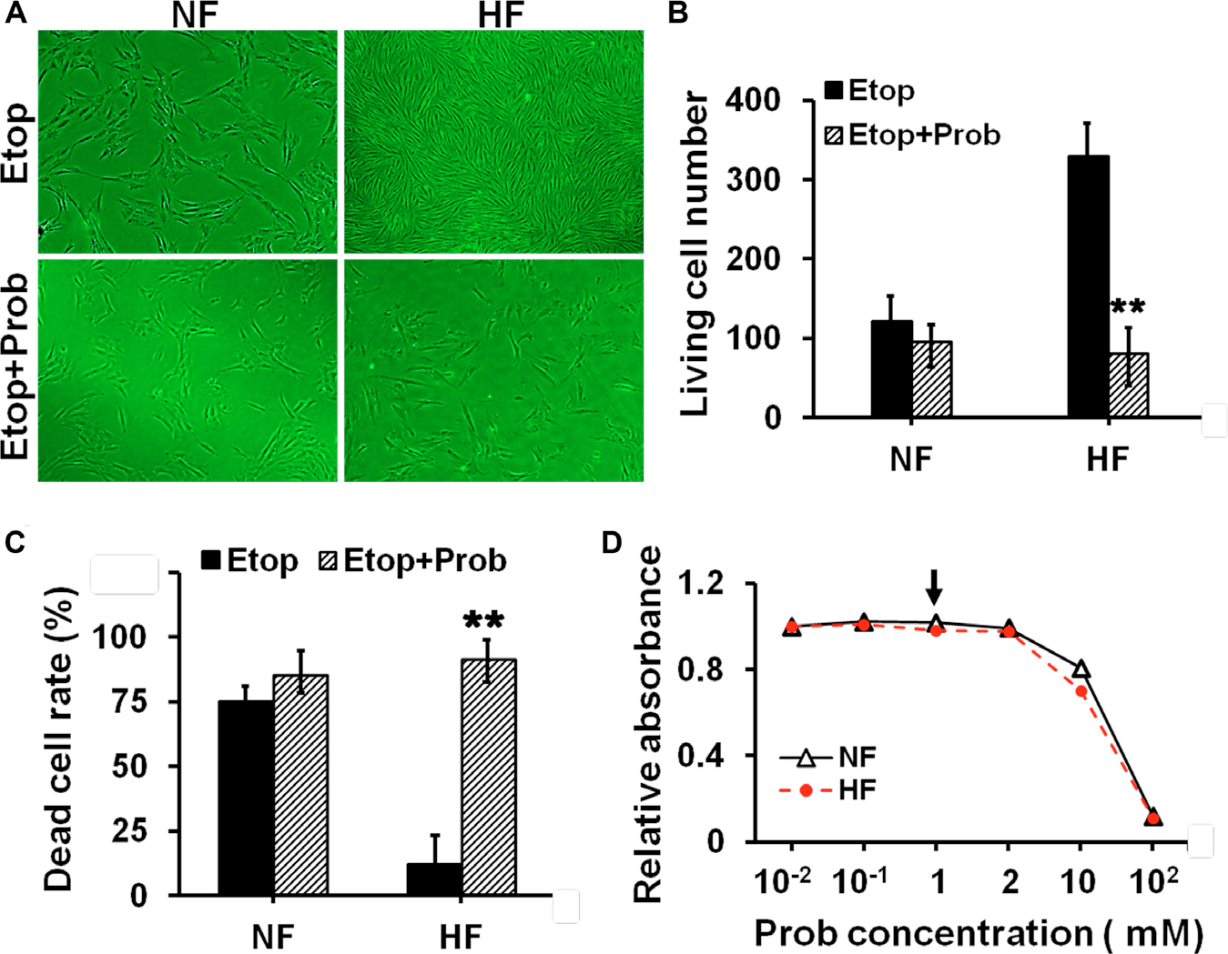
Evaluation of the resistance to etoposide phosphate after pre-treating cells with MRP1 inhibitor in NF and HF (**A**) Cell density in NF and HF after Etop alone or with probenecid (Prob) pre-treatment was visualized under light microscope. (**B**) The living cell number under each treatment in (A) was counted and compared. (**C**) The dead cell rate in each treatment group was analyzed by flow cytometry and compared. Error bars represent means ± SD (*n* = 4), **p < 0.01. (**D**) The cytotoxicity of Prob in NF and HF was determined by MTT assay with the absorbance measured at 540 nm. Prob at 5 μM was the concentration used in this study as indicated by black arrow.

Next we tried to elucidate whether drug resistance in HS was related to the overexpression of P-glycoprotein or MRP1. PSC833, an inhibitor for P-glycoprotein, and probenecid (Prob), an inhibitor for MRP1, were selected and used in this study. Cells were pre-treated with PSC833 or DMSO (vehicle control) in Figure 4, or pre-treated with Prob or DMSO (vehicle control) in Figure 5 for 12 h, and then subjected to Ver or Etop treatment for 12 h. Light microscopy and flow cytometry analysis revealed that PSC833 (Figure 4) or Prob (Figure 5) pre-treatment significantly attenuated drug resistance in HF, causing rapid decrease on living cell number (Figures 4A–4B and 5A–5B) and increase on dead cell rate (Figures 4C and 5C). The cytotoxicity of PSC833 and Prob at different concentrations was measured by MTT assay. PSC833 at 5 μM (Figure 4D) and Prob at 1 mM (Figure 5D), the concentrations that did not cause cellular toxicity, were used in this study.

### The association between P-glycoprotein/MRP1 and actin was up-regulated in hypertrophic scar fibroblasts

**Figure 6:**
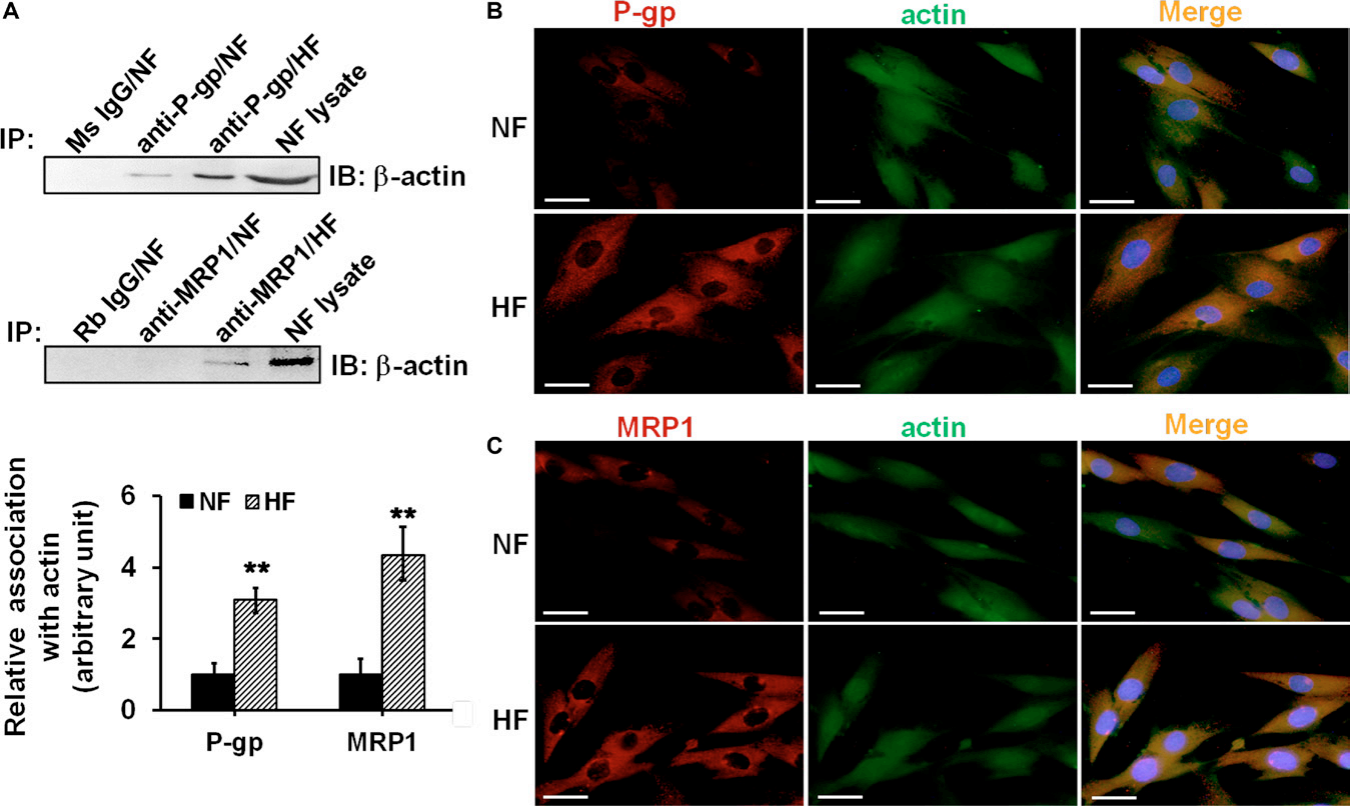
Study to assess the association and co-localization between P-glycoprotein/MRP1 with actin in NF and HF (**A**) Co-IP was performed to assess the association between P-glycoprotein/MRP1 with actin in NF and HF. Mouse (Ms) or rabbit (Rb) IgG was used as negtive control for its corresponding primary antibody. Lysate from NF without Co-IP was used as positive control. The relative association of P-glycoprotein/MRP1 with actin in NF and HF was summarized and compared in histogram. Error bars represent means ± SD (*n* = 4), **p < 0.01. (**B**–**C**) Dual-labeled immunofluorescent staining was used to assess the co-localization between P-glycoprotein (b, *red*) or MRP1(c, *red*) with actin (*green*). Nuclei were visualized with DAPI (*blue*). The co-localization was indicated in *orange* in merged images. Scale bar: 20 μm.

The ultimate question was to reveal the underlying mechanisms that confer HS drug resistance. Recent studies have indicated the involvement of P-glycoprotein-cytoskeletal protein (*e.g*. actin) association in drug transporting protein functions [11]. Therefore, we moved to examine whether the interaction between P-glycoprotein/MRP1 and actin existed in skin fibroblasts, and whether this interaction was different between NF and HF. Co-IP analysis showed NF exhibited weak interaction beetween P-glycoprotein/MRP1 and actin, which was significantly up-regulated in HF (Figure 6A). Dual-labelled immunofluorescent analysis further showed remarkably more co-localization of P-glycoprotein/MRP1 with actin in HF than that in NF (Figure 6B–6C).

### The disruption of P-glycoprotein/MRP1-actin association by latrunculin-A abrogated drug resistance in hypertrophic scar fibroblasts

**Figure 7:**
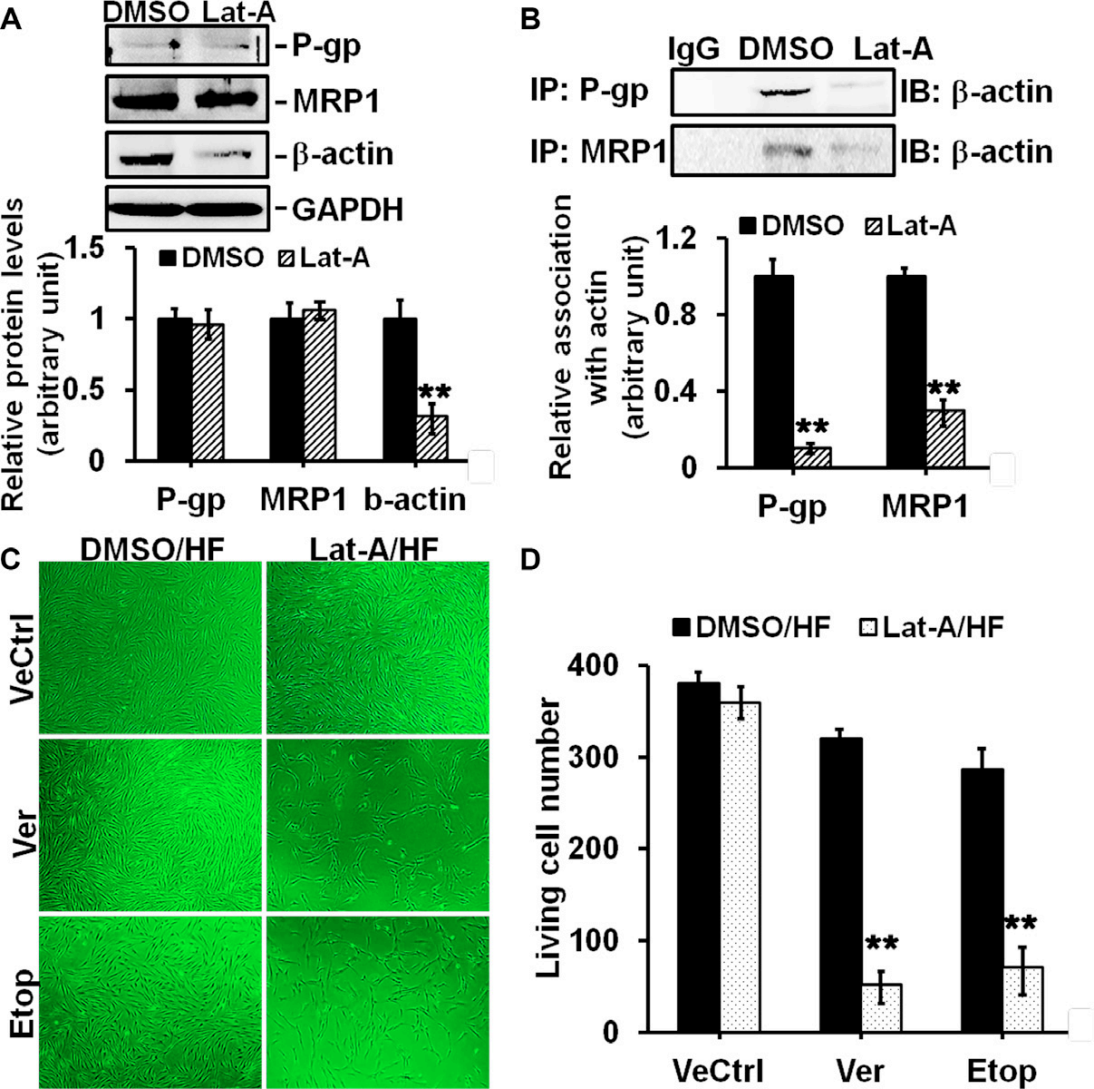
Effects of latrunculin-A on P-glycoprotein/MRP1-actin association and on drug resistance in HF (**A**) Immunoblotting to assess the effect of latrunculin-A (Lat-A) on protein level of P-glycoprotein, MRP1 or β-actin in HF. GAPDH served as an equal protein loading control. (**B**) Co-IP to assess the effect of Lat-A on P-glycoprotein/MRP1-actin association in HF. Normal mouse or rabbit IgG served as negtive control. (**C**) Cell density in HF after Ver or Etop treatment with/without Lat-A pre-treatment was visualized under light microscope. (**D**) The living cell number in each treatment group was counted and compared. Error bars represent means ± SD (*n* = 6), **p < 0.01.

We explored whether disrupting P-glycoprotein/MRP1-actin association would affect drug resistance in HS. HF were treated with latrunculin-A, an actin depolymerization agent, or DMSO for 12 h and lysed for immunoblotting. Results showed that latrunculin-A did not affect the protein level of P-glycoprotein or MRP1, while it significantly decreased β-actin level by 70% (Figure 7A). Notably, Co-IP analysis showed that latrunculin-A disrupted the strong interaction of P-glycoprotein/actin by ~90% and MRP1/actin by ~70% in HF (Figure 7B). When HF were pre-treated with latrunculin-A for 12 h and then subjected to Ver or Etop for another 12 h, the living cell number was reduced from ~300 to ~50 when compared to Ver or Etop treatment alone (Figure 7C–7D), suggesting latrunculin-A-induced disruption of P-glycoprotein/MRP1-actin association abolished the drug resistance in HF.

### The use of specific anti-actin antibody abrogated drug resistance in hypertrophic scar fibroblasts

**Figure 8:**
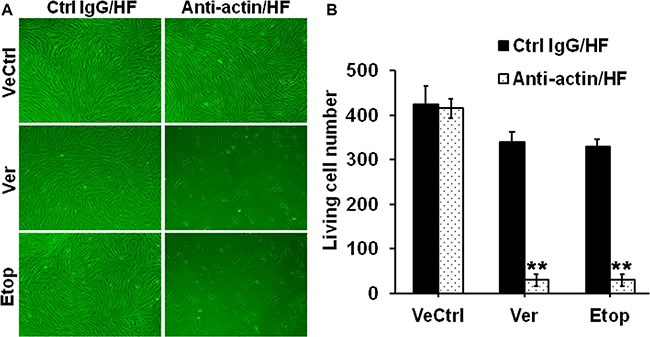
Effect of the specific anti-actin antibody on drug resistance in HF (**A**) Cell density in HF after Ver or Etop treatment with/without the anti-actin antibody pre-treatment was visualized under light microscope. (**B**) The living cell number in each treatment group was counted and compared. Error bars represent means ± SD (*n* = 6), **p < 0.01.

An anti-actin antibody was used to specifically inhibit actin function. Briefly, HF were pre-treated with the anti-actin or normal rabbit IgG (vehicle control) for 24 h and then subjected to Ver or Etop treatment for 12 h, the living cell number was significantly reduced compared to Ver or Etop treatment alone (Figure 8A–8B), suggesting that the inhibition of actin function abolished drug resistance in HF.

## DISCUSSION

HS is featured by the overgrowth of dermal fibroblasts [12] and considered as a benign skin tumor. Thus, chemotherapy has been used for HS treatment. Drug resistance is one of the major clinical challenges in cancer chemotherapy and often leads to the failure of chemotherapy and cancer recurrence [1–2]. Given the similarity between HS and tumor, it is reasonable to expect the existence of drug resistance phenomenon in HS or HS-derived fibroblasts. In this study, verapamil and etoposide phosphate, two agents with potential anti-cancer effect, were selected to investigate drug resistance in HS. As shown in Figure 1, normal skin fibroblasts were more sensitive to verapamil and etoposide, and exhibited less living cell number (Figure 1A–1B) and higher dead cell rate (Figure 1C–1D) than hypertrophic scar fibroblasts, suggesting the presence of drug resistance in HS.

Drug transporters play important roles in mediating the chemosensitivity and drug resistance in cancer cells [13]. P-glycoprotein and MRP1 are the two best characterized drug resistance-related transporters responsible for the failure of cancer chemotherapy [14]. They are often over-expressed in cancer cells and actively reduce intracellular drug accumulation below toxic concentration via pumping drugs out of cells [15–16]. Importantly, the resistance to verapamil or etoposide has been reported to be mediated by P-glycoprotein or MRP1, respectively [17]. Our study revealed significantly elevated expression of P-glycoprotein and MRP1 in scar fibroblasts, while only slight expression in normal fibroblasts (Figure 2B). Interestingly, although P-glycoprotein and MRP1 were reported to primarily locate at the plasma membrane in many epithelial and endothelial cells [18], our study showed that P-glycoprotein and MRP1 were predominantly distributed in the cytoplasm in cultured scar fibroblasts, with tiny staining in normal fibroblast cytoplasm (Figure 2A). *In vivo* studies further confirmed above observation, results showed that although normal skin dermis expressed little P-glycoprotein and MRP1, their protein levels were notably enhanced in hypertrophic scar dermis (Figure 3C). IHC on normal dermis and scar dermis revealed the tissue distribution of P-glycoprotein and MRP1. Due to the diversity on fibroblast density in normal dermis and scar dermis, the positivley-stained fibroblast rate was compared between two groups instead of the positively-stained cell number, results indicated significantly higher stained cell rate in scar dermis than that in normal dermis (Figure 3A–3B). Above data, on the other hand, demonstrate the existence of drug resistance in HS. Besides, PepT1, an oligopeptide transporter that does not confer drug resistance, showed no difference in its expression and distribution between normal skin and HS and served here as an internal control (Figures 2, 3).

Inhibiting the function of drug transporter is a way to study drug resistance [19–20]. In order to elucidate whether the resistance to verapamil and etoposide in HS was confered by P-glycoprotein and MRP1, PSC833 and probenecid were used to specifically inhibit the function of P-glycoprotein and MRP1 in cultured fibroblasts. Results showed that the resistance to verapamil or etoposide in scar fibroblasts was remarkably abolished when cells was pre-treated with PSC833 or probenecid. (Figures 4, 5). Also, pre-treating cells with PSC-833 or probenecid significantly decreased the living cell number (Figures 4A–4B and 5A–5B) and increased the dead cell rate (Figures 4C–4D and 5C–5D) in scar fibroblasts, while did not affect normal fibroblasts. These data demonstrate that drug resistance in HS is mediated by P-glycoprotein and MRP1.

There are emerging evidences linking the function of drug transporters, such as P-glycoprotein, with actin microfilament system. A few reports have suggested the involvement of P-glycoprotein-actin association in drug resistance [21–22]. A recent study showed that cytochalasin, an actin-perturbing agent, enhanced intracellular drug accumulation in leukemia cells, indicating the attenuation of drug resistance in these cells [23]. Another similar study reported the disruption of actin stress-fiber network increased the intracellular drug accumulation and cytotoxic effect in osteosarcoma cells, suggesting the abrogation of drug resistance [9]. Our present study confirmed the structural interaction between P-glycoprotein and actin filament in skin fibroblasts by co-immunoprecipitation and immuno-colocalization, and for the first time revealed the association between MRP1 and actin (Figure 6). More importantly, the association and co-localization between P-glycoprotein/MRP-1 and actin were significantly elevated in scar fibroblasts than those in normal fibroblasts (Figure 6), suggesting a potential linkage between actin cytoskeletal network and the resistance in scar fibroblasts. When scar fibroblasts were treated with latrunculin-A, an actin cytoskeleton disrupting compound, only the steady-state level of β-actin was affected, but not P-glycoprotein and MRP1, suggesting the specificity of this inhibitor (Figure 7A). Also, latrunculin-A significantly abolished the association between P-glycoprotein/MRP1 and actin in scar fibroblasts (Figure 7B). When cells were pre-treated with latrunculin-A, the resistance to verapamil or etoposide in scar fibroblasts was remarkably attenuated (Figure 7C–7D). Moreover, the use of specific anti-actin antibody abrogated drug resistance in hypertrophic scar fibroblasts (Figure 8A–8B). These data make it conceivable that the linkage of drug transporter with actin cytoskeleton functions as a potential mechanism in the development of drug resistance in HS.

Taken together, this study reveals the presence of drug resistance in HS that is closely related to the enhanced expression of drug resistance-related transporters, such as P-glycoprotein and MRP1, defines for the first time an enhanced association between P-glycoprotein/MRP1 and actin cytoskeleton in HS, and also discloses a dramatic reduction of P-glycoprotein/MRP1-mediated drug resistance when actin function was specifically inhibited. Research on new molecules or gene therapy strategies aiming at selectively disrupting drug transporter-cytoskeleton interaction or P-glycoprotein/MRP1-mediated drug resistance, may stem from the results of this study to improve the efficacy of HS chemotherapy.

## MATERIALS AND METHODS

### Ethics statement

The protocol for studies using human samples was reviewed and approved by the Institutional Ethics Committee of the Fourth Military Medical University. Patients who offered skin samples including normal skin and hypertrophic scar provided their written, informed consent. Patient samples were deidentified and anonymized prior to analysis.

### Primary skin fibroblast isolation and culture

Normal skin and hypertrophic scar tissues were obtained from sixteen patients (Table 1) who had been undergoing plastic surgery in Department of Burns and Cutaneous Surgery, Xijing Hospital (Xi’an, China). All specimens were collected with the permission from the Human Subjects Committee of the local institution. Dermal portions were minced and incubated in a solution of collagenase type I (Sigma-Aldrich, St. Louis, MO) at 0.1 mg/ml at 37°C for 3 h to separate fibroblasts. Isolated fibroblasts were then pelleted and grown in DMEM (Gibco, Grand Island, NY) supplemented with 10% fetal calf serum (Gibco), 100 U/ml penicillin, and 100 U/ml streptomycin at 37°C in a humidified atmosphere with 5% (v/v) CO_2_. Cells were seeded into six-well plates at 2 × 10^5^ cells/well in normal growth medium and cells at the third and fourth sub-passages were used for experiments. Prior to drug treatment, cells were cultured in serum-free DMEM for 12 h and then treated with various drugs (detailed chemical information in Table 2). Cells in control group were added with an equal volume of serum-free medium with corresponding vehicle control. Cells were harvested and analyzed at time points indicated in each experiment.

**Table 1:** The profile of each sample for primary culture

Sample	Gender	Age (years)	Biopsy site	Duration of lesion (months)	Etiology
NF/HF 1	Male	17	Cheek	10	Post-operation
NF/HF 2	Male	20	Arm	16	Electric injury
NF/HF 3	Male	20	Shoulder	6	Burn
NF/HF 4	Male	21	Shoulder	5	Scald
NF/HF 5	Male	24	Shoulder	24	Scald
NF/HF 6	Male	36	Buttock	7	Burn
NF/HF 7	Male	38	Chest	17	Post-operation
NF/HF 8	Male	42	Chest	15	Post-operation
NF/HF 9	Female	16	Chest	8	Trauma
NF/HF 10	Female	18	Chest	14	Scald
NF/HF 11	Female	24	Shoulder	9	Burn
NF/HF 12	Female	25	Arm	10	Scald
NF/HF 13	Female	27	Cheek	12	Burn
NF/HF 14	Female	43	Shoulder	11	Burn
NF/HF 15	Female	45	Back	10	Trauma
NF/HF 16	Female	46	Back	18	Scald

Abbreviations: NF, normal skin fibroblast; HF, hypertrophic scar-derived fibroblast.

**Table 2:** Detailed information on the chemicals used in this study

Chemicalnames	Function	Vendor	Cat no.	Lot no.	Working concentration	Solution
verapamil	P-glycoprotein substrate	Santa cruz biotechnology	sc-3590	152-11-4	200 μM	ethanol
PSC833	P-glycoprotein inhibitor	Santa cruz biotechnology	sc-361298	121584-18-7	5 μM	DMSO
etoposide phosphate	MRP1 substrate	Santa cruz biotechnology	sc-357357	117091-64-2	50 μM	cold water
probenecid	MRP1 inhibitor	Santa cruz biotechnology	sc-202773	57-66-9	1 mM	DMSO
latrunculin-A	actin depolymerization reagent	Santa cruz biotechnology	sc-202691	76343-93-6	0.2 μM	DMSO

### Immunoblotting

Briefly, 50 μg lysates from normal skin dermis, hypertrophic scar dermis, and their corresponding fibroblasts were subject to SDS-PAGE and transferred onto PVDF membranes (Millipore, Bedford, MA). After blocking with 5% non-fat milk, membranes were incubated with specific primary antibodies (Table 3) at 4°C overnight. On the following day, membranes were washed and incubated with corresponding HRP-conjugated secondary antibodies at 37°C for 1 h. Proteins were visualized by enhanced chemiluminescence system using Fluor Chem FC (Alpha Innotech).

**Table 3:** Summary of primary antibodies used in this study

Antigen	Cat no.	Host	Vendor	Dilution				
				IB	IHC	IF	IP	Inhibitor
P-glycoprotein	sc-55510	Mouse	Santa cruz biotechnology	1:200	1:50	1:50	1:40	
MRP1	ab24102	Mouse	Abcam	1:200		1:50		
MRP1	sc-13960	Rabbit	Santa cruz biotechnology		1:50		1:40	
PepT1	sc-20653	Rabbit	Santa cruz biotechnology	1:200	1:50			
Actin	sc-1616	Goat	Santa cruz biotechnology	1:500				
Actin	4967	Rabbit	Cell signaling technology			1:100		1:500

Abbreviations: IB, immunoblotting; IHC, immunohistochemistry; IF, immunofluorescence; IP, immunoprecipitation.

### Co-immunoprecipitation (Co-IP)

Lysates from normal skin fibroblasts and hypertrophic scar fibroblasts were prepared in IP lysis buffer (10 mM Tris, 0.15 M NaCl, 1% NP-40, and 10% glycerol, pH 7.4 at 22°C) supplemented with protease and phosphatase inhibitor cocktails according to manufacturer's instruction. Co-IP was performed to identify if actin was structurally associated with P-glycoprotein or MRP1. In brief, 2 μg mouse or rabbit IgG was added to 400 μg lysates of fibroblasts and incubated for 1 h before precipitated with 10 μl protein A/G agarose beads (Santa Cruz Biotechnology, Santa Cruz, CA) for 1 h, supernatant was then obtained after centrifuging at 1000 *g* for 5 min. This pre-cleaning step removed non-specific interacting proteins from cell lysates. Thereafter, lysates were incubated with 2 μg normal mouse/rabbit IgG as negative control or specific anti-P-glycoprotein or anti-MRP1 antibody for Co-IP on a Labnet MiniLabRoller overnight, to be followed by incubation with 20 μl protein A/G agarose beads to extract the immunocomplexes. Thereafter, beads were washed with IP lysis buffer and immunocomplexes (P-glycoprotein/MRP1 and its interacting protein partners) were extracted in SDS sample buffer at 100°C for SDS-PAGE and immunoblot analysis. 50 μg normal fibroblast lysates without IP served as the positive control.

### Immunocytofluorescence

Fibroblasts were fixed in 4% paraformaldehyde for 10 min. After washing with PBS, cells were permeabilized with 0.1% Triton-X 100 for 10 min and blocked with 1% bovine serum albumin at room temperature for 1 h. Samples were then incubated with 1:50 diluted primary antibody at 4°C overnight at appropriate dilutions (Table 3). After being washed with PBS, samples were incubated with 1:100 diluted FITC-488 or Cy3-555 conjugated secondary antibody (Invitrogen, Carlsbad, CA) at 37°C for 1 h. After being washed with PBS three times, samples were stained with DAPI for 30 min. Coverslips were then mounted onto glass slides and viewed with Olympus Box-Type Photofluorography Unit Model FSX100 (Olympus, Japan).

### Immunohistochemistry

Hypertrophic scar and normal skin tissues were fixed in 10% buffered formalin solution overnight. The 4-μm thickness paraffin-embedded sections were dewaxed, the endogenous peroxidase activity was quenched with 3% hydrogen peroxide for 15 min, and then blocked for non-specific bindings by normal goat serum for 30 min. Sections were then incubated overnight at 4°C with appropriate primary antibody dilutions (Table 3) and then followed by the application of PV-6000 Histostain TM Kit (ZSJQ, China). Briefly, sections were incubated with biotinylated secondary antibody, streptavidin–biotin–horseradish peroxidase was used for signal amplification and diaminobenzidine (DAB) for staining, sections were then counterstained with hematoxylin. Negative control was achieved by an isotypematched IgG in each of the immunostaining. Images were captured using an Olympus fluorescence microscope with an Olympus FSX100 digital camera. All images were subsequently compiled and analyzed using IPP (Image-Pro Plus) software package.

### Flow cytometry

Cell death was evaluated by flow cytometry. Cells were treated with various drugs for specific period and washed with PBS. After washing, cells were resuspended in 100 μl Annexin-V (diluted at 1:100 in 1% BSA in PBS) added with 5 μl FITC plus 5 μl PI, and incubated for 15 min at room temperature in dark. 400 ul Annexin-V was added to above mixture, cell death was then measured by BD FACSAria III flow cytometry (BD Bioscience, San Jose, CA).

### Cytotoxicity assay

The cytotoxicity of PSC833 and probenecid was evaluated by MTT assay. In brief, fibroblasts were seeded in 96-well plates at 3 × 10^4^ cells/well and kept overnight allowing full attachment. On the next day culture medium was replaced with fresh one supplemented with various concentrations of probenecid at 10^−2^, 10^−1^, 1, 2, 10 and 10^2^ mM or PSC833 at 0.05, 0.5, 5, 10, 50 and 500 μM, cells were allowed to grow for 48 h. Then 100 μl MTT (10 mg/ml) was added to each well 4 h before completion of this incubation. Thereafter, 100 μl DMSO was added to each well. The optical density (OD) was measured at 540 nm with a Bio-Rad Model 680 Plate Reader (Bio-Rad Laboratories, Hercules, CA).

### Statistical analysis

Each experiment reported herein was repeated at least three to four times, excluding pilot experiments. Statistical analysis was performed using the GB-STAT software package (version 7.0; Dynamic Microsystems, Silver Spring, MD). One-way ANOVA was performed and followed by Dunnett's test for multiple comparisons. In selected experiments, Student's *t* test was used for paired comparisons.
